# Supplementary material for: G Protein-Coupled Receptor 40 Agonist LY2922470 Alleviates Ischemic-Stroke-Induced Acute Brain Injury and Functional Alterations in Mice
Source: Int J Mol Sci. 2023 Jul 31;24(15):12244. doi: 10.3390/ijms241512244 (PMC10418587; doi:10.3390/ijms241512244)
Supplement: Supplementary file 1 [file ijms-24-12244-s001.zip › ijms-2463946-supplementary.pdf]

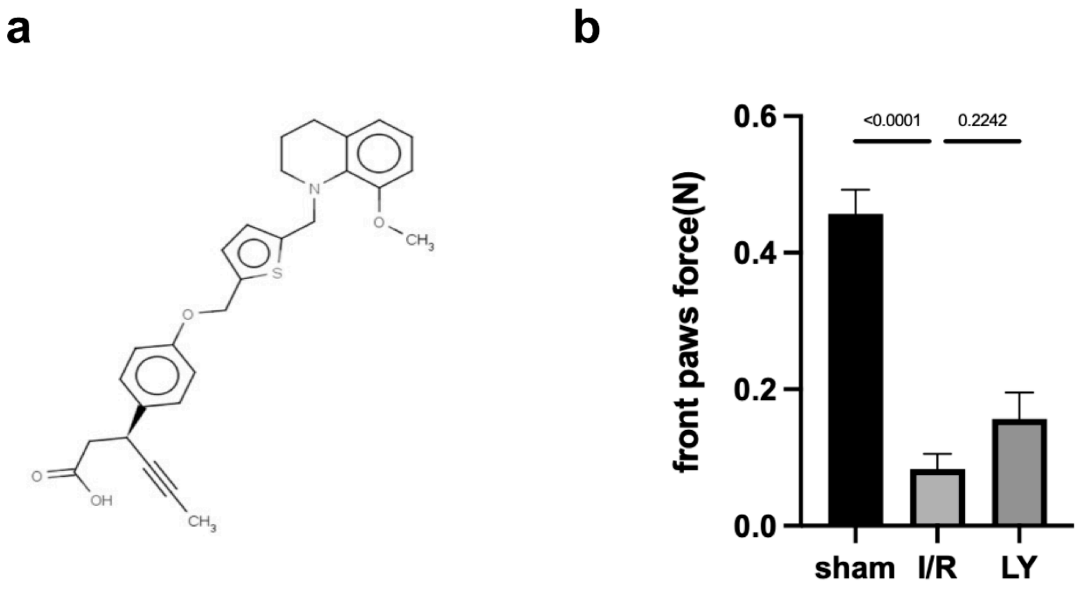

Supplementary Figure S1. (a) Chemical structure diagram of LY2922470. (b) Data statistics of mouse forelimb grip strength after 24h of reperfusion. Data are mean ± SEM from the indicated number of biological replicates (sham, LY group, *n* = 12; IR groups, *n*=11).
